# Hepatitis A Outbreak Associated with Cuba — Florida, June 2024–February 2026

**DOI:** 10.15585/mmwr.mm7537a1

**Published:** 2026-09-24

**Authors:** Natnari Natalie Linwong, Marie K. Etienne, Anthoni Llau, Scot Menke, Shannon Patelsky, Natalia Cano, Timothy J. Doyle

**Affiliations:** ^1^Bureau of Epidemiology, Florida Department of Health; ^2^Epidemic Intelligence Service, CDC; ^3^Miami-Dade County, Florida Department of Health, Miami, Florida; ^4^Palm Beach County, Florida Department of Health, West Palm Beach, Florida; ^5^Broward County, Florida Department of Health, Fort Lauderdale, Florida; ^6^Bureau of Public Health Laboratories, Florida Department of Health; ^7^Division of State and Local Readiness, Office of Readiness and Response, CDC.

**Summary**

**What is already known about this topic?**

Cuban news media have reported increased numbers of hepatitis A cases since 2024; however, official government reports about hepatitis A in Cuba have been limited.

**What is added by this report?**

During June 1, 2024–February 28, 2026, among 249 hepatitis A cases reported in Florida, 76 (31%) were associated with recent travel to Cuba. Among 18 genotyped cases, 15 shared a matching genotype IA strain, consistent with a common origin.

**What are the implications for public health practice?**

Counseling on preventive measures including hepatitis A vaccination and immune globulin should be offered to travelers before planned trips to Cuba. Providers should consider hepatitis A among patients with clinically compatible symptoms who have recently been in Cuba.

## Abstract

Beginning in early 2025, Florida Department of Health (FDOH) staff members noted an unusually high proportion of reported hepatitis A cases among travelers who had recently been in Cuba. Hepatitis A outbreaks have been reported in Cuban local news media; however, government reports are limited. FDOH investigated to characterize hepatitis A cases associated with travel to Cuba among Florida residents (including four patients who had recently emigrated from Cuba). FDOH found that among 249 confirmed hepatitis A cases identified during June 2024–February 2026, 76 (31%) patients reported recent travel to Cuba before symptom onset. Among patients with Cuba travel–associated hepatitis A, the median age was 39 years, 70% were male, 87% were White, and 92% were Hispanic or Latino. Patients lived in 13 (19%) of Florida’s 67 counties. Among 29 patients who completed supplemental travel questionnaires, 55% had visited Havana; other travel destinations included eight additional Cuban provinces. Hepatitis A virus (HAV) genotyping of 18 isolates from unique patients with outbreak-associated cases identified 15 (83%) isolates that matched HAV genotype IA outbreak strain 1, two (11%) that matched IB strain 1, and one (6%) that matched IA strain 2. These findings are consistent with what would be expected were widespread hepatitis A transmission affecting multiple regions of Cuba. CDC recommends hepatitis A vaccination or receipt of immune globulin before travel to Cuba. Providers’ consideration of hepatitis A among patients returning from Cuba who have hepatitis A–compatible signs and symptoms, together with elicitation of a detailed travel history, might aid diagnosis of Cuba travel–associated cases.

## Investigation and Results

### Reports of Hepatitis A in Cuba

Since 2024, Cuban news media have reported hepatitis A outbreaks in multiple provinces related to drinking water contamination associated with disruptions in sanitation and utility systems (*1*,*2*). However, official Cuban national surveillance data have not been publicly disseminated. In December 2024, the Ministry of Public Health of Cuba issued a tourist alert regarding increased cases of hepatitis A (*3*). In early 2025, FDOH observed that a high proportion of recent hepatitis A cases reported in Florida occurred among patients who had traveled to Cuba during their exposure periods (15–50 days before symptom onset), and in August 2025, the U.S. Embassy in Cuba issued a health alert regarding increased numbers of cases in Havana (*4*).

### Florida’s Cuban-American Population

Florida is home to approximately 1.8 million Cuban Americans, who account for 8% of the state’s population (*5*). Many Florida residents travel to Cuba regularly to visit family and friends. Considering these connections and the limited availability of information regarding the hepatitis A outbreaks in Cuba, FDOH investigated to characterize cases associated with travel to Cuba.

### Public Health Investigation of Hepatitis A Cases in Florida

Hepatitis A is a reportable condition in Florida. FDOH uses the national surveillance case definition for hepatitis A. FDOH staff members investigate all reported hepatitis A cases by interviewing patients and reviewing medical records to obtain demographic information, document the clinical course, identify risk factors for infection (e.g., recent history of travel, incarceration, housing status, substance use, sexual activity, and consumption of certain foods), and identify close contacts for possible prophylaxis and follow-up.

### HAV Genotyping

For all patients whose cases were reported and met the hepatitis A surveillance case definition, medical providers and commercial laboratories were asked to forward available blood samples to FDOH’s Bureau of Public Health Laboratories (BPHL) for HAV genotyping. BPHL performed genotyping based on the 349-nucleotide VP1-P2B junction region of the HAV genome (*6*). Next-generation sequencing was conducted on specimens, and data were uploaded to CDC’s Global Hepatitis Outbreak Surveillance Technology (GHOST) platform* and compared with an HAV genetic sequence reference set to identify matching strains (*7*).

### Cuba Travel–Associated Outbreak Case Definition

FDOH created an outbreak case definition for hepatitis A cases associated with travel to Cuba. A Cuba travel–associated hepatitis A outbreak case was defined as a hepatitis A infection in a Florida resident meeting the surveillance case definition for confirmed hepatitis A, with symptom onset during June 1, 2024–February 28, 2026, and who met at least one of the following criteria: 1) known history of travel to or emigration from Cuba 15–50 days before symptom onset, 2) gene sequencing data indicating genotype IA outbreak strain 1, or 3) epidemiologic linkage to a confirmed outbreak case. The gene sequencing criterion was included because FDOH observed that several cases associated with travel to Cuba had matching genotype IA strain 1, which had not previously been identified in Florida or reported in GHOST, and which suggested a common epidemiologic origin.

### Supplemental Questionnaire Data

In addition to the routine epidemiologic investigation conducted for all hepatitis A cases in Florida, additional travel information was obtained for patients with cases meeting the Cuba travel–associated outbreak case definition in the three southeastern counties with the highest numbers of outbreak-associated cases (Broward, Miami-Dade, and Palm Beach). These three counties accounted for 59 (75%) of the 79 cases that met the outbreak case definition. FDOH staff members in these counties obtained additional details from patients regarding travel to Cuba using a standardized, supplemental, telephone-administered questionnaire. Patients were asked about their method of travel, where and with whom they stayed, sources of drinking water, and whether they had heard reports of hepatitis A while in Cuba. Among the 59 patients from the three most affected counties, 29 (49%) patients completed the supplemental questionnaire; the others either refused or could not be reached. Questionnaires were administered during December 2025–February 2026 to patients whose cases had previously been reported and investigated as well as to patients with newly reported cases. This activity was reviewed by CDC, deemed not research, and conducted consistent with applicable federal law and CDC policy.^†^

### Characteristics of Patients with Hepatitis A

A total of 249 confirmed cases of hepatitis A with illness onset during June 1, 2024–February 28, 2026, were reported to FDOH. Among these, 118 (47%) patients reported international travel during their exposure period, including 76 (31%) who had traveled to Cuba. Three additional cases were linked to the outbreak through gene sequencing data or epidemiological linkage. Overall, 79 cases (32%) met the outbreak case definition. Among patients with outbreak-associated hepatitis A, the median age was 39 years, 70% were male, 87% were White, and 92% were Hispanic or Latino; 72% were hospitalized and no deaths occurred (Table). Outbreak-associated cases occurred among residents of 13 counties in Florida; 46 (58%) of the 79 patients who met the outbreak case definition lived in Miami-Dade County (Figure 1).

**TABLE:** Characteristics of hepatitis A cases associated with Cuba — Florida, June 2024–February 2026

Characteristic (no. with available information)	No. (%)
**Age, yrs (79)**	
Median (IQR)	39 (31–51)
**Sex (79)**	
Female	24 (30.4)
Male	55 (69.6)
**Race (78)**	
Asian	1 (1.3)
Black or African American	2 (2.6)
White	68 (87.2)
Other	7 (9.0)
**Ethnicity (78)**	
Hispanic or Latino	72 (92.3)
Not Hispanic or Latino	6 (7.7)
**Emergency department visit (79)**	
Yes	60 (75.9)
No	19 (24.1)
**Hospitalized (78)**	
Yes	56 (71.8)
No	22 (28.2)
**Genotype and strain (18)**	
IA outbreak strain 1	15 (83.3)
IB strain 1	2 (11.1)
IA strain 2	1 (5.6)
**Length of stay in Cuba, days (64)**	
Median (IQR)	11 (6–26)
**Mode of travel (29)**	
Commercial flight	23 (79.3)
Charter flight	6 (20.7)
Province of overnight stay (29)*	
La Habana	16 (55.2)
Villa Clara	3 (10.3)
Matanzas	3 (10.3)
Mayabeque	2 (6.9)
Pinar del Río	2 (6.9)
Sancti Spíritus	2 (6.9)
Holguín	1 (3.4)
Camagüey	1 (3.4)
Granma	1 (3.4)
**Persons traveler stayed with (28)**	
Only family or friends	26 (92.9)
Both family or friends and hotels or guesthouses	2 (7.1)
Drinking water sources while in Cuba (29)*	
Tap	12 (41.4)
Bottled	20 (69.0)
Ice	4 (13.8)
Boiled or filtered	4 (13.8)
**Heard reports of hepatitis A while in Cuba (29)**	
Yes	13 (44.8)
No	16 (55.2)

* Because multiple responses were allowed, the number of responses does not match the number of respondents.

**FIGURE 1:**
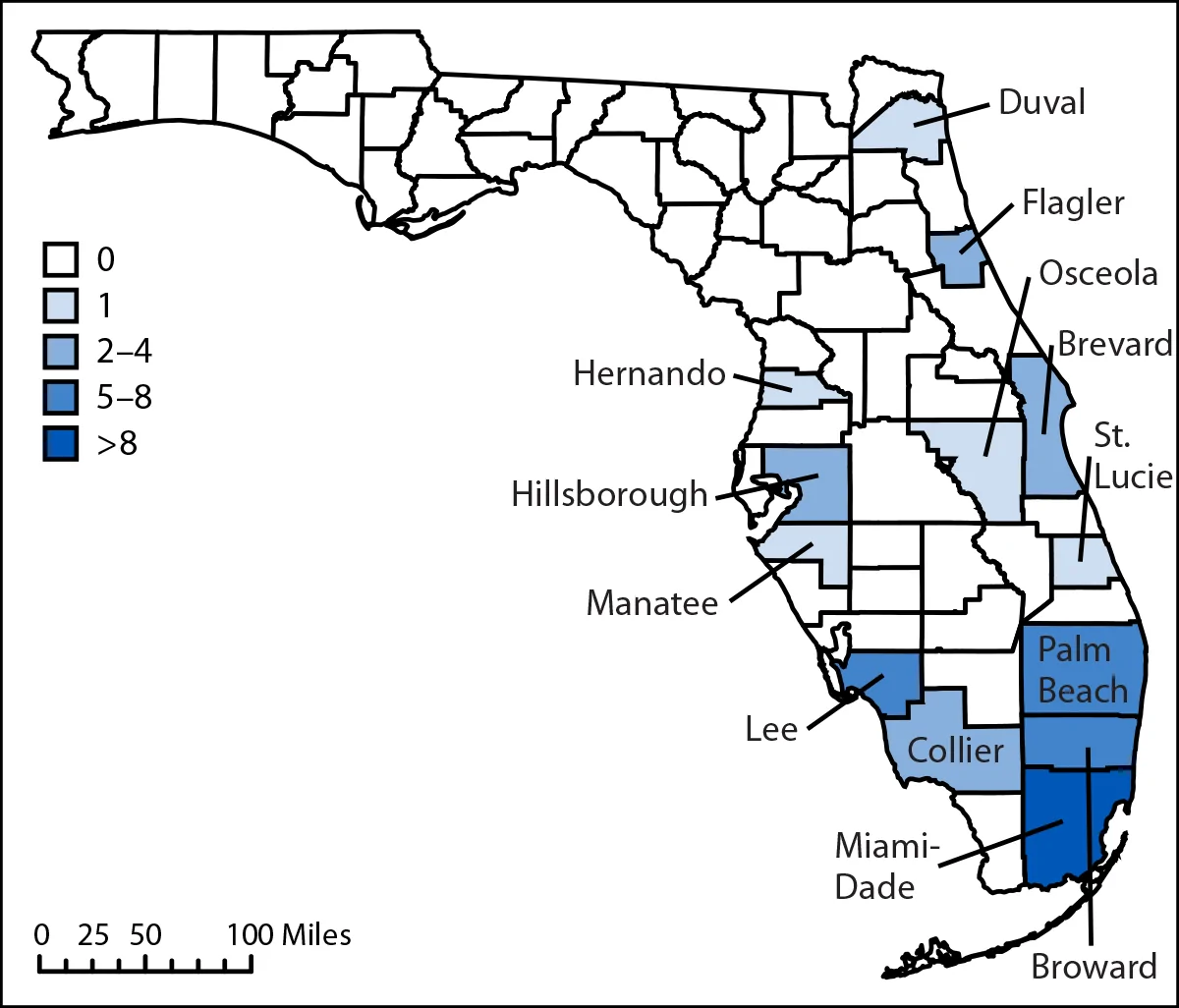
Number of outbreak-associated hepatitis A cases* among Florida residents recently in Cuba, by county — June 2024–February 2026 * The total number of outbreak-associated cases per county were Brevard, two; Broward, five; Collier, two; Duval, one; Flagler, two; Hernando, one; Hillsborough, three; Lee, six; Manatee, one; Miami-Dade, 46; Osceola, one; Palm Beach, eight; and St. Lucie, one. The number of cases associated with the dominant hepatitis A virus genotype IA outbreak strain 1 were one each in Brevard, Lee, and Osceola counties, and six each in Miami-Dade and Palm Beach counties.

### History of Travel to Cuba

Among the 79 outbreak-associated cases, 64 (81%) patients had recently visited Cuba with known dates of travel, eight (10%) had recent travel to Cuba during their exposure periods but with exact travel dates unknown, and four (5%) had recently emigrated from Cuba. Two (3%) patients who could not be interviewed had an unknown travel history but were included based on gene sequencing data that matched the predominant HAV strain associated with travel to Cuba. One (1%) patient had no recent travel history but was epidemiologically linked to a confirmed case in a household contact in Florida who had traveled to Cuba. No other epidemiologic linkages were identified among cases. Among 64 patients with known travel dates, the median length of stay in Cuba was 11 days (IQR = 6–26 days). Seven of 76 (9%) patients reported that while in Cuba, each was exposed to a family member who had received a hepatitis A diagnosis. Risk factors for HAV infection in addition to travel to Cuba were identified among three patients: two male patients (3%) reported having had sex with men, and one patient (1%) reported having had household contact with another patient in Florida who had traveled to Cuba. No patients reported experience with homelessness, incarceration, or recreational drug use during their potential exposure period.

Supplemental questionnaires provided additional details regarding travel to Cuba. A total of 16 of 29 respondents (55%) stayed in Havana, and 14 (48%) stayed in other provinces (one stayed in both Havana and another province). Of 28 respondents who provided additional information about where they stayed, 26 (93%) stayed only with family and friends. Twelve (41%) of 29 respondents reported drinking tap water, 20 (69%) reported drinking bottled water, four (14%) reported drinking boiled or filtered water, and four (14%) reported consuming ice (several respondents reported that they consumed water from multiple sources). Overall, 13 (45%) of 29 respondents had heard reports of hepatitis A cases or outbreaks from family or friends while in Cuba.

### Gene Sequencing

Gene sequencing was completed for isolates from 18 outbreak-associated cases^§^ from five counties (Brevard, Lee, Miami-Dade, Osceola, and Palm Beach). Of these cases, 15 (83%) matched HAV genotype IA outbreak strain 1, two (11%) matched genotype IB strain 1, and one (6%) matched IA strain 2 (Figure 2).

**FIGURE 2:**
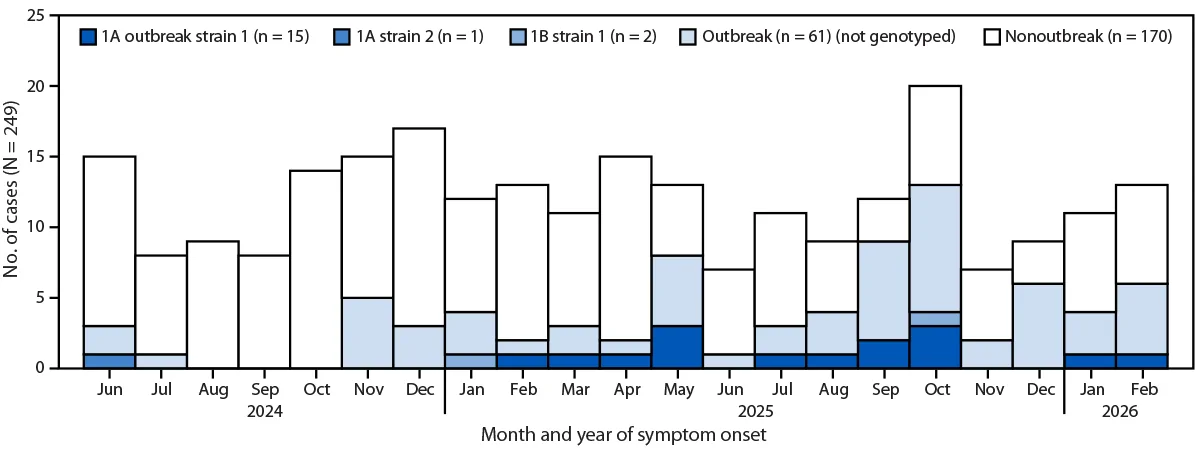
Number of hepatitis A cases, by month of symptom onset and strain — Florida, June 2024–February 2026

## Discussion

During 2025, Florida public health officials noticed that numerous hepatitis A cases were occurring among persons with recent travel to Cuba. Because of the large Cuban-American population in Florida, an investigation was conducted that found that 76 of 249 (31%) reported hepatitis A cases during June 2024–February 2026 were associated with travel to Cuba; the investigation connected three additional cases with the outbreak by matching genotype or epidemiologic linkage. Among outbreak cases for which gene sequencing data were available, 83% matched genotype IA outbreak strain 1, which was first reported in GHOST and identified in an outbreak-associated case in Florida in February 2025. Two additional HAV strains were observed: a second genotype IA strain in a single case, and an additional HAV strain in two cases identified as genotype IB, indicating no common chain of transmission from the other genotyped cases. The gene sequences from cases associated with travel to Cuba did not match any sequence patterns observed in Florida before this outbreak. Sequence patterns from Cuba were not available.

The high proportion of hepatitis A cases linked to travel from a single country during the analysis period differs from historical patterns in Florida. During 2010–2017, 34% of hepatitis A cases in Florida were associated with international travel, compared with 47% observed in the analysis period, and 32% associated with Cuba. During 2018–2019, a period characterized by a large hepatitis A outbreak associated with person-to-person transmission, only 3.3% of cases were associated with international travel, and cases with international travel were more likely to be associated with unique HAV nucleotide sequence patterns, rather than matching strains indicative of a common origin (*6*).

These findings indicate that an outbreak of hepatitis A in Cuba, occurring over several months, has affected Florida residents with recent travel history to Cuba. The proportion of hepatitis A cases among Florida residents reporting travel to Cuba exceeds historical norms, and among nearly all patients with history of travel to Cuba, no other risk factors for HAV infection were identified. Except for one episode of household transmission, no other evidence of secondary spread in Florida was found.

The risk for hepatitis A among travelers to Cuba is ongoing. Additional cases in Florida residents have been identified since March 2026, with exposures in Cuba as recently as August 2026.

### Limitations

The findings in this report are subject to at least four limitations. First, whereas 79 outbreak-associated cases were reported in 13 counties, sequencing data were only available for 18 cases (23%), all of which occurred among residents of five counties. Second, supplemental questionnaires were completed for 37% of outbreak-associated cases from three counties; therefore, sequencing and questionnaire data might not be representative of all outbreak-associated cases. Third, supplemental questionnaires were administered up to 18 months after illness and responses might have been subject to recall bias. Finally, because the exposure period for most patients included time spent in both Cuba and Florida, it cannot be confirmed that all HAV infections were acquired in Cuba.

### Implications for Public Health Practice

This investigation found evidence of travel-associated hepatitis A among Florida residents who had recently returned from Cuba, where news media have reported increased numbers of hepatitis A cases since 2024. Based on these findings, eliciting a detailed travel history from patients with symptoms clinically compatible with hepatitis A and consideration of the possibility of hepatitis A among patients with recent travel history to Cuba might help identify cases. For travelers receiving health care before traveling to Cuba, providers should review CDC recommendations for hepatitis A vaccination and consider immune globulin for high-risk travelers (adults aged >40 years, immunocompromised persons, and persons with chronic liver disease) departing within 2 weeks (*8*,*9*). Counseling may also be provided regarding risk factors and preventive measures, including food and water safety and hand hygiene.

## Footnotes

* CDC developed the GHOST system to characterize viral genotypes and transmission links among cases with hepatitis C virus infection in outbreak settings; the content is also applicable to hepatitis A infections in outbreak settings. Molecular Epidemiology of HCV Infection | Viral Hepatitis | CDC

† 45 C.F.R. part 46, 21 C.F.R. part 56; 42 U.S.C. Sect. 241(d); 5 U.S.C. Sect. 552a; 44 U.S.C. Sect. 3501 et seq.

§ Sixteen of these 18 cases occurred among the 76 persons who reported travel to Cuba; the other two cases occurred among persons who had unknown travel history but met the outbreak case definition because their genotyping results matched the dominant outbreak strain.
